# Supplementary material for: Phylogeny and Historical Biogeography of Veronica Subgenus Pentasepalae (Plantaginaceae): Evidence for Its Origin and Subsequent Dispersal
Source: Biology (Basel). 2022 Apr 21;11(5):639. doi: 10.3390/biology11050639 (PMC9138021; doi:10.3390/biology11050639)
Supplement: Supplementary file 1 [file biology-11-00639-s001.zip › biology-1674672-supplementary.pdf]

Table S1. Details of specimens of *Veronica* included in this study, including locality, herbarium information and GenBank accession numbers.

No.	Taxon name	Locality	Herbarium (Voucher)	Collector	ITS GenBank
1	*V. acrotheca 1*	Iran: Lorestan, SE of Dorud, lake Gahar	*MIR 3975*	M. Mirtadzadini et al.	ON202639
2	*V. acrotheca 2*	Iran: Azarbaijan, Arasbaran, Ilan-khosh	*TUH 35457*	A. Ghahreman et al.	ON202640
3	*V. antalyensis*	Turkey: Antalya, Qazipasa, Cimbiti Yaylas	*E 00326080*	H. Sumbul	ON202641
4	*V. aragonensis 1*	Spain: Huesca, collado de Ceresa, Peña Montañesa	*SALA 121537*	A. Abad de Blas et al.	KT361667
5	*V. aragonensis 2*	Spain: Granada, Huéscar, Sierra de la Sagra	*SALA 93528*	J. A. Sanchez-Agudo et al.	KT361668
6	*V. aragonensis 3*	Spain: Huesca, Turbón massif, between the Turbonet and Aligas peak	*SALA 121536*	M. M. Martinez-Ortega	AY741517
7	*V. armena 1*	Turkey: Erzurum, Mt. Palandöken	*MA 687629*	A. Herrero et al.	KT361709
8	*V. armena 2*	Cult. NYBG	*WU 1404*	Struwe	AF313040
9	*V. aucheri 1*	Iran, Central Alborz		399	ON202642
10	*V. aucheri 2*	Iran: Mazandaran, Mt. Damavand	*MIR 3622*	M. Doostmohammadi	ON202643
11	*V. austriaca subsp. jacquinii 1*	Botanischer Garten Bonn, cultivated	*BONN*	D. C. Albach	AF313000
12	*V. austriaca subsp. jacquinii 2*	Montenegro: Treskavac Mts. between Borkovici and Boricje	*SALA 149369*	B. M. Rojas-Andres et al.	KT361675
13	*V. austriaca subsp. jacquinii 3*	Bulgaria: Stara Zagora, Nova Mahala, near Nikolaev	*SALA 149377*	B. M. Rojas-Andres et al.	KT361676
14	*V. austriaca subsp. jacquinii 4*	Croatia: Josipdol, between Oštarije and Ribaric´i	*SALA 149042*	B. M. Rojas-Andres et al.	KJ630596
15	*V. austriaca subsp. jacquinii 5*	Bosnia: Travnik, Vlašic´	*SALA 149389*	B. M. Rojas-Andres et al.	KT361677
16	*V. austriaca subsp. dentata 1*	Austria: Krems, between Weissenkirchen and Dürnstein	*SALA 149043*	B. M. Rojas-Andres et al.	KJ630593
17	*V. austriaca subsp. dentata 2*	Austria: Wien, Kalksburg	*SALA 149383*	B. M. Rojas-Andres et al.	KT361671
18	*V. baranetzkii*		-	-	ON202644
19	*V. bogosensis*	Russia: North Ossetia, Ingushetia and Chechnya	*MO*	Prima	AF486359
20	*V. bombycina subsp. bolkardaghensis*	NYBG, cultivated	*WU 1406*	Struwe	AF486358
21	*V. bombycina subsp. bombycina*	NYBG, cultivated	*WU 1403*	Struwe	AF486353
22	*V. bombycina subsp. froediniana*	Turkey: Pelli Dagi, south of Kuzgunkiran pass.	*WU 0027040*	D. C. Albach and F. Ozgokce 709	KT361710
23	*V. caespitosa 1*	Turkey: Kastamonu, Ilgaz Daglari, 35 km south of Kastamonu	*E 01005965*	J. R. Edmondson	ON202645
24	*V. caespitosa 2*	Turkey: Ulu Dag near Bursa	*E 01005968*	J. J. F. E. De Wilde	ON202646

25	*V. caucasica 1*	Georgia: Kazbegi	*WU 326*	D. C. Albach	AF486357
26	*V. caucasica 2*	Cultivated	57		ON202647
27	*V. chionantha*	Iran: Alborz, 2 km east of Azadbar to Gachsar	*MIR 3616*	M. Doostmohammadi	ON202648
28	*V. cinerea*	RBG Kew, cultivated	*K 113*	D. C. Albach and M. Chase	AY144458
29	*V. crinita 1*	Bulgaria: Varna, between Vinitsa and Aldza monastery	*SALA 149037*	B. M. Rojas-Andres et al.	KJ630594
30	*V. crinita 2*	Bosnia: Ravan Planina, Mt. Tajan	*SALA 149244*	B. Frajman	KT361669
31	*V. crinita 3*	Bulgaria: Plodiv, near Popovitsa	*SALA 149038*	B. M. Rojas-Andres et al.	KT361670
32	*V. cuneifolia 1 subsp. isaurica*	NYBG, cultivated	*WU 1409*	Struwe	AF486354
33	*V. cuneifolia 2*	Turkey: Antalya, Akseki, west of Taslica	*1148*	Abdel	ON202649
34	*V. cuneifolia 3*	Turkey: Mt. Tahtali	*OLD 1159*	D. C. Albach	ON202650
35	*V. czerniakows 1*	Iran: Khorassan: Kopet-Dagh	*IRAN 39177*	F. Terme	AF486362
36	*V. czerniakows 2*	Iran: Khorassan, north of Shirvan, Golul Sarani P. A.	*MIR 3629*	M. Doostmohammadi and A. Talebi	ON202651
37	*V. dalmatica 1*	Croatia: Dubrovnik, Gromacˇa	*SALA 149039*	B. M. Rojas-Andres et al.	KJ630595
38	*V. dalmatica 2*	Montenegro: Žabljak	*SALA 149287*	B. M. Rojas-Andres et al.	KT361674
39	*V. daranica 1*	Iran: Esfahan, Boein, 6 km north of Hosseinabad	*MIR 3546*	M. Doostmohammadi	ON202652
40	*V. daranica 2*	Iran: Bakhtiari, SW of Samsami, Mt. Mili	*MIR 3970*	M. Mirtadzadini	ON202653
41	*V. dichrus*	NYBG, cultivated	*WU 1407*	Struwe	AF312998
42	*V. elmaliensis*	Turkey: Lydia: Mt. Sipyli	*E 01005953*	J. Bornmuller	ON202654
43	V. farinosa	Iran: Esfahan, Semirom, Surmandeh Mt.	TUR 213948	Alava	AY741518
44	*V. fragilis 1*	Iran: Chaharmahal, Sartange-Mahmoud	*MIR 3544*	M. Doostmohammadi	ON202655
45	*V. fragilis 2*	Iran: Zagros Mountain		358	ON202656
46	*V. fridericae 1*	Turkey: Van, north of Gedikbasi		610	ON202657
47	*V. fridericae 2*	Turkey: Van, between Baskale and Güzelsu	E 00326073	Sorger and Buchner	ON202658
48	*V. fuhsii*	Turkey		863	ON202659
49	*V. gaubae 1*	Iran: Semnan, Mt. Neyzeva	*MIR 3625*	M. Doostmohammadi	ON202660
50	*V. gaubae 2*	Iran: Mazandaran, Alasht, Mt. Eltamer	*MIR 3631*	M. Doostmohammadi and A. Ghorbanalizadeh	ON202661
51	*V. khorassanica 1*	Iran: Semnan, Shahrud, NW of Mojen waterfall	*MIR 3976*	M. Mirtadzadini	ON202662
52	*V. khorassanica 2*	Iran: Khorassan, Ghorkhod, west of Bash-Kalate	*FUMH 45195*	M. Joharch and F. Memeriani	ON202663
53	*V. kindlii*	North Macedonia: Gevgelija, Mt. Kozuf	*SALA 149278*	B. M. Rojas-Andres et al.	KT361689
54	*V. kopetdaghensis*	Iran: Khorasan, NW of Mashhad, Hezar-Masjed peak	*FUMH 44208*	M. Joharchi and M. Behroozian	ON202664

55	*V. krylovii 1*	Russia: Rep. Altai, Ulaganskii ,vicin. of lake Choibekkel	*ALTB*	A. I. Schmakov et al.	KT361681
56	*V. krylovii 2*	Kazakhstan: Ridge Tarbagataj	*ALTB*	S. Smirnov et al.	KT361682
57	*V.kurdica subsp. filicaulis 1*	Iran: Bakhtiari, SW of Samsami, Mt. Mili	*MIR 3969*	M. Mirtadzadini	ON202665
58	*V. kurdica subsp. filicaulis 2*	Iran: Kerman, western slopes of Mt. Palvar	*MIR 3596*	M. Doostmohammadi	ON202666
59	*V. kurdica subsp.kurdica 1*	Iran: Mazandaran, south of Kelardasht		1740	ON202667
60	*V. kurdica subsp. kurdica 2*	Iran: Ghazvin, west of Abe-garm, Kise-jin to Dashtak	*MIR 3547*	M. Doostmohammadi	ON202668
61	*V. kurdica subsp. kurdica 3*	Iran: east of Damavand, around lake Tar	*MIR 3971*	M. Mirtadzadini	ON202669
62	*V. kurdica subsp. kurdica 4*	Iran: Firuzkuh to Polur, between Najafdar and Laasem	*MIR 3978*	M. Mirtadzadini	ON202670
63	*V. leiocarpa*	Jordan: between Al-Rishah and Rahma	*OLD1310*	D. C. Albach	KJ630597
64	*V. liwannensis*	NYBG, cultivated	*WU 1411*	Struwe	AF312997
65	*V. macrostachya 2 (subsp. sorgerae)*	Turkey: Icel, Silifke-Gulnar, 17 km south of Silifke	*E 00074752*	M. Nydegger	ON202671
66	*V. mazanderanae 1*	Iran: Gilan, Javaherdasht		1741	ON202672
67	*V. mazanderanae 2*	Iran: Mazandaran, between Firukuh and Qaemshahr, Gaduk	MIR 3655	*M. Mirtadzadini*	ON202673
68	*V. microcarpa 1*	Azerbaijan, Sadarak		873	ON202674
69	*V. microcarpa 2*	Turkey: Hakkari, near Bagisli		2606	ON202675
70	*V. microcarpa 3*	Iran: Azarbaijan, Khoy to Qotur	*TUH 34576*	American-Iranian Botanical Deligation	ON202676
71	*V. minuta*			799	ON202677
72	*V. mirabilis*	Iran, Mazandaran, Alasht, Mt. Eltamer	*MIR 3632*	M. Doostmohammadi and A. Ghorbanalizadeh	ON202678
73	*V. multifida 1*	Turkey: prov. Antalya, Taurus	*OLD 1143*	D. C. Albach	KT361711
74	*V. multifida 2*	Turkey: Erzurum, Uzundere	-	-	DQ534899
75	*V. multifida 3*	Turkey: Erzurum, Uzunkavak, south of Serdarli		2603	ON202679
76	*V. multifida 4*	Iran: Ardebil, SE of Khalkhal	*MIR 3610*	M. Doostmohammadi	ON202680
77	*V. multifida 5*	Iran: Mazandaran, Ramsar, before Garasmasar	*MIR 3613*	M. Doostmohammadi and A. Ghorbanalizadeh	ON202681
78	*V. oltensis*	NYBG, cultivated	*WU 1405*	Struwe	AF312995
79	*V. orbiculata 1*	Croatia: Peljesak, between Trstenik and Pijavicˇino	*SALA 149294*	B. M. Rojas-Andres et al.	KT361683
80	*V. orbiculata 2*	Croatia: between Omis and Makarska, Brela	*SALA 149337*	B. M. Rojas-Andres et al.	KT361684
81	*V. robiculata 3*	Bosnia: Mostar, Mt. Hum	*SALA 149336*	B. M. Rojas-Andres et al.	KT361685
82	*V. orbiculata 4*	Bosnia: Mostar, Mt. Hum	*SALA 149336*	B. M. Rojas-Andres et al.	KT361686

83	*V. orientalis 1*	Turkey: Van, Karabel pass	*WU 701*	D. C. Albach	AY741515
84	*V. orientalis 2*	Iran: Esfahan, NW of Semirom to Vanak	*MIR 4006*	M. Mirtadzadini	ON202682
85	*V. orientalis 3*	Turkey: Van, Gevas	*OLD*	1738	ON202683
86	*V. orientalis 4*	*Turkey: Van, north of Muradiye*	*OLD*	2607	ON202684
87	*V. orientalis 5*	*Turkey: Van, north of Ozalp, near Yukaribalcikli*	*OLD*	2605	ON202685
88	*V. orientalis 6*	*Lebanon: Nabatiyeh, Baraachit*	*OLD*	1315	ON202686
89	*V. orientalis 7*	*Turkey: Adiyaman, Kahta, Camlica*	*OLD*	1302	ON202687
90	*V. orientalis 8*	*Georgia: north of Vardisubani, Sheerteba*	*OLD*	2608	ON202688
91	*V. orientalis 9*	Iran: Fars, between Dashte-Arjan and Khaneh-Zenian	*MIR 3989*	M. Mirtadzadini and R. Naderi	ON202689
92	*V. orientalis 10*	Iran, Hamedan, Avaj	*TUH 16922*	F. Attar and F. Dadjou	JF409918
93	*V. orientalis 11*	Turkey: Van, south of Guzelsu, Murataldi	*OLD*	2604	ON202690
94	*V. orsiniana 1*	Italy: Abruzzo, La Majella	*SALA 149297*	E. Rico et al.	KT361687
95	*V. orsiniana 2*	Italy: Abruzzo, La Majella	*SALA 149298*	E. Rico et al.	KT361688
96	*V. paederotae 1*	Iran: Mazandaran, Mt. Alam-kuh	*MIR 3636*	M. Doostmohammadi and A. Ghorbanalizadeh	ON202691
97	*V. paederotae 2*	Iran: Mazandaran, Mt. Azad kuh	*WU 7901*	Klein	AF509783
98	*V. parsana*	Iran: Ardebil, east of Abi-beiglu, Asbine mt.	*MIR 3608*	M. Doostmohammadi	ON202692
99	*V. pectinata 1*	Turkey: Bolu, south of Abant Golu	*MA 688478*	C. Aedo et al.	KT361712
100	*V. pectinata 2*	NYBG, cultivated	*WU 1410*	Struwe	AY144460
101	*V. peduncularis 1*	Georgia: Mtskheta, monastery Sedaseni	*SALA 110319*	M. M. Martinez-Ortega and al	KT361714
102	*V. peduncularis 2*	Georgia: Kazbegi	*WU 325*	D. C. Albach	AF486356
103	*V. polifolia 1*	Jordan: Aqaba, north of Al-Rishah	*OLD1309*	D. C. Albach	KJ630601
104	*V. polifolia 2*	Lebanon: Nabatiyeh, Baraashit		1316	ON202693
105	*V. polium 1*	Turkey: Pelli Dagi, south of Kuzgunkiran pass.	*WU 0025732*	D. C. Albach and F. Ozgokce 712	KT361713
106	*V. polium 2*	Turkey: Bitlis: Pelli Dag	*E 01016482*	P. H. Davis	ON202694
107	*V. prostrata 1*	Austria: Rohrendorf bei Krems, Saubühel	*SALA 149040*	B. M. Rojas-Andres et al.	KJ630602
108	*V. prostrata 2*	Switzerland: Valais, Charrat	*SALA 149312*	B. M. Rojas-Andres et al.	KT361690
119	*V. prostrate 3*	Bulgaria: Shumen, Madara	*SALA 149317*	B. M. Rojas-Andres et al.	KT361691
110	*V. rhodopea 1*	Bulgaria: Pazardzhik, Belmeken, near the	*SALA 149321*	B. M. Rojas-Andres et al.	KT361692
111	*V. rhodopea 2*	Bulgaria: Mt. Rila	*SOM*	Bondev	AY144459

112	*V. rosea 1*	Morocco: Ifrane, Azrou, Djebel Hebri	*SALA 149323*	D. Pinto-Carrasco et al.	KT361693
113	*V. rosea 2*	Algeria: Tlemcen, Col de Krorchef	*SALA 149324*	A. Juan-Galladro et al.	KT361694
114	*V. rosea 3*	Morocco: Midelt, Great Atlas, Cirque de Jaffar	*SALA 121638*	L. Delgado-Sanches et al.	AY741519
115	*V. satureiifolia 1*	France: Dep. Lozère, Aven Armand	*SALA 149356*	B. M. Rojas-Andres et al.	KT361695
116	*V. satureiifolia 2*	Spain: Huesca, Ansó, Linza, Paso del'Onso	*SALA 124593*	L. Delgado-Sanches and M. M. Martinez-Ortega	KT361696
117	*V. satureiifolia 3*	Germany: Baden Württemberg, Bopfingen, Rohrbachmühle	*SALA 124594*	M. M. Martinez-Ortega	KT361697
118	*V. schizostegia = V. macrostachya1 (ssp. schizostegia)*	Iran: Kordestan, Auraman valley	*MIR 3568*	M. Mirtadzadini and F. Attar	ON202695
119	*V. sennenii 1*	Spain: Álava, Salinas de Añana, way up to the collado de la Rastrilla	*SALA 149394*	B. M. Rojas-Andres et al.	KT361698
120	*V. sennenii 2*	Spain: Cantabria, Sonabia	*SALA 149395*	B. M. Rojas-Andres et al.	KT361699
121	*V. taurica*	Ukrain: Alushta: north side of Roman Kosh.	*E 01005980*	Davis	ON202696
122	*V. tauricola 1*	Turkey: Nigde, Ala Dag	*E 01005973*	P. W. Wood and W. B. Gibson	ON202697
123	*V. tauricola 2*	Turkey: Sivas, east flank of Tahtali Dag	*E 01005974*	J. M. Watson	ON202698
124	*V. tenuifollia subsp. fontqueri 1*	Spain: Málaga, Sierra de las Nieves, Puerto de los Pilones	*MGC 46659*	F. J. Hernandez-Garcia et al.	KT361672
125	*V. tenuifollia subsp. fontqueri 2*	Spain: Almería, Sierra de Gádor, Llanos de Boliches	*SALA 95041*	F. J. Hernandez-Garcia et al.	KT361673
126	*V. tenuifolia subsp. javalambrensis 1*	Spain: Salamanca, La Mata de la Armuña	*SALA 149328*	B. M. Rojas-Andres and N. Lopez-Gonzalez	KT361679
127	*V. tenuifolia subsp. javalambrensis 2*	Spain: Valdelinares, El Hornillo	*SALA 110650*	E. Rico and X. Giraldez	KT361680
128	*V. tenuifolia subsp. tenuifolia 1*	Spain: Navarra, Cáseda, at intersection of road NA534 and Canal de Bárdenas	*SALA 95040*	L. Delgado-Sanches and M. M. Martinez-Ortega	KT361700
129	*V. tenuifolia subsp. tenuifolia 2*	Spain: Huesca, Arro, near the road to Los Molinos	*SALA 93496*	L. Delgado-Sanches and M. M. Martinez-Ortega	KT361701
130	*V. tenuifolia subsp. tenuifolia 3*	Spain: Huesca, Arro.	*SALA 93496*	M. M. Martinez-Ortega	AY741516
131	*V. teucrioides 1*	Greece: Kozani, Mt. Siniátsikon, over Námata	*SALA 149270*	A. Herrero et al.	KT361702
132	*V. teucrioides 2*	Greece: Mt. Olympus	*SALA 149330*	B. M. Rojas-Andres et al.	KT361703
133	*V. teucrium var. teucrium 1*	Bulgaria: 3 km east of Tran, road to Pernik	*SALA 149044*	B. M. Rojas-Andres et al.	KJ630603
134	*V. teucrium var. teucrium 2*	Germany: Nordrhein-Westfalen, Euskirchen, between Iversheim and Arloff	*SALA 149414*	A. Abad de Blas et al.	KT361704

135	*V. teucrium var. angustifolia 1*	France: Dep. Haute-Savoie, Mt. Salève	*SALA 149399*	B. M. Rojas-Andres et al.	KT361705
136	*V. teucrium var. angustifolia 2*	France: Dep. Eure et Loir, Châteaudun, Thiville	*SALA 149413*	A. Abad de Blas et al.	KT361706
137	*V. thymoides*	Cultivated		203	ON202699
138	*V. turrilliana 1*	Turkey: Vize, toward Kömürköy-Alkpinar	*SALA 149333*	B. M. Rojas-Andres et al.	KT361707
139	*V. turrilliana 2*	Bulgaria: 15 km north of Malko Turnovo, near the bridge on the river Veleka	*SALA 149334*	B. M. Rojas-Andres et al.	KT361708
140	*V. turrilliana 3*	Turkey: Istanbul	*WU 0023358*	D. C. Albach 278	AF486360
141	*V. vendetta-deae*	Georgia: Kazbegi	*WU 327*	D. C. Albach	AF486361
Outgroups					
142	*V. chamaedrys*	RBG Kew	*K*	M. Chase	AF313003
143	*V. polita*	Spain: Salamanca, Ciudad Rodrigo	*SALA 149255*	J. A. Sánchez-Agudo	KT361716
144	*V. campylopoda*	Jordan: near Ruin Shorbek	*WU 4152*	P. Schonswetter and A. Tribsch	AF486364
